# Supplementary material for: Characterization of the Monkeypox Virus [MPX]-Specific Immune Response in MPX-Cured Individuals Using Whole Blood to Monitor Memory Response
Source: Vaccines (Basel). 2024 Aug 26;12(9):964. doi: 10.3390/vaccines12090964 (PMC11436000; doi:10.3390/vaccines12090964)
Supplement: Supplementary file 1 [file vaccines-12-00964-s001.zip › vaccines-3136225-supplementary.pdf]

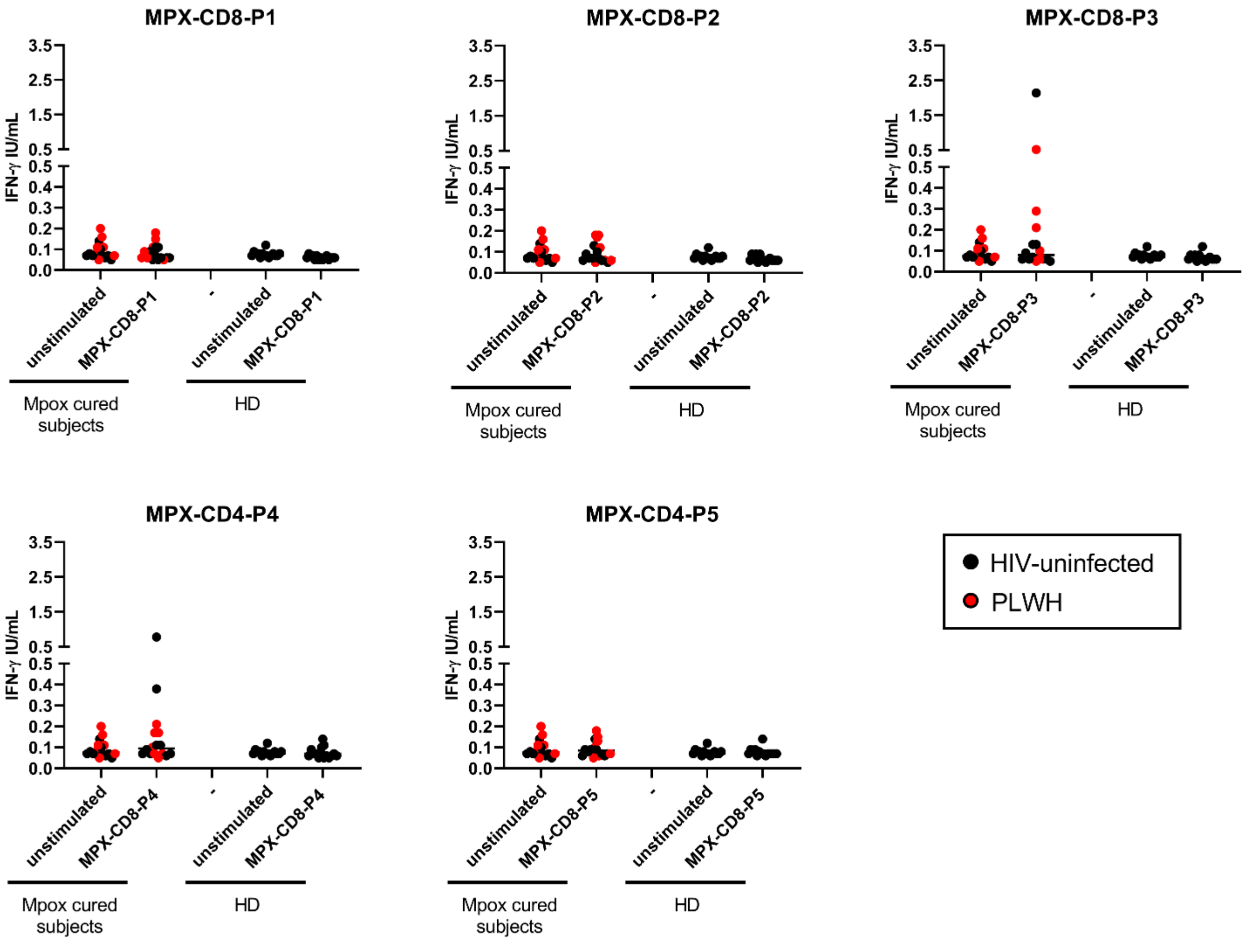

Figure S1: MPX-CD8-specific T cell response from in vitro stimulated samples from Mpox-cured individuals. Graphs report the IFN-γ response induced by MPX-CD8-P1; MPX-CD8-P2; MPX-CD8-P3, MPX-CD8-P4, MPX-CD8-P5 stimulation. ELISA was performed in plasma samples from whole blood stimulation and IFN-γ expressed as IU/mL; the IFN-γ value of the stimulated conditions were not subtracted by the unstimulated control value. The horizontal lines represent the median; statistical analysis was performed using the Wilcoxon test; black plots refer to HIV-uninfected subjects, red plots refer to PLWH. Footnotes: IFN-γ: interferon-γ; HD: healthy donor; PLWH: people living with HIV.

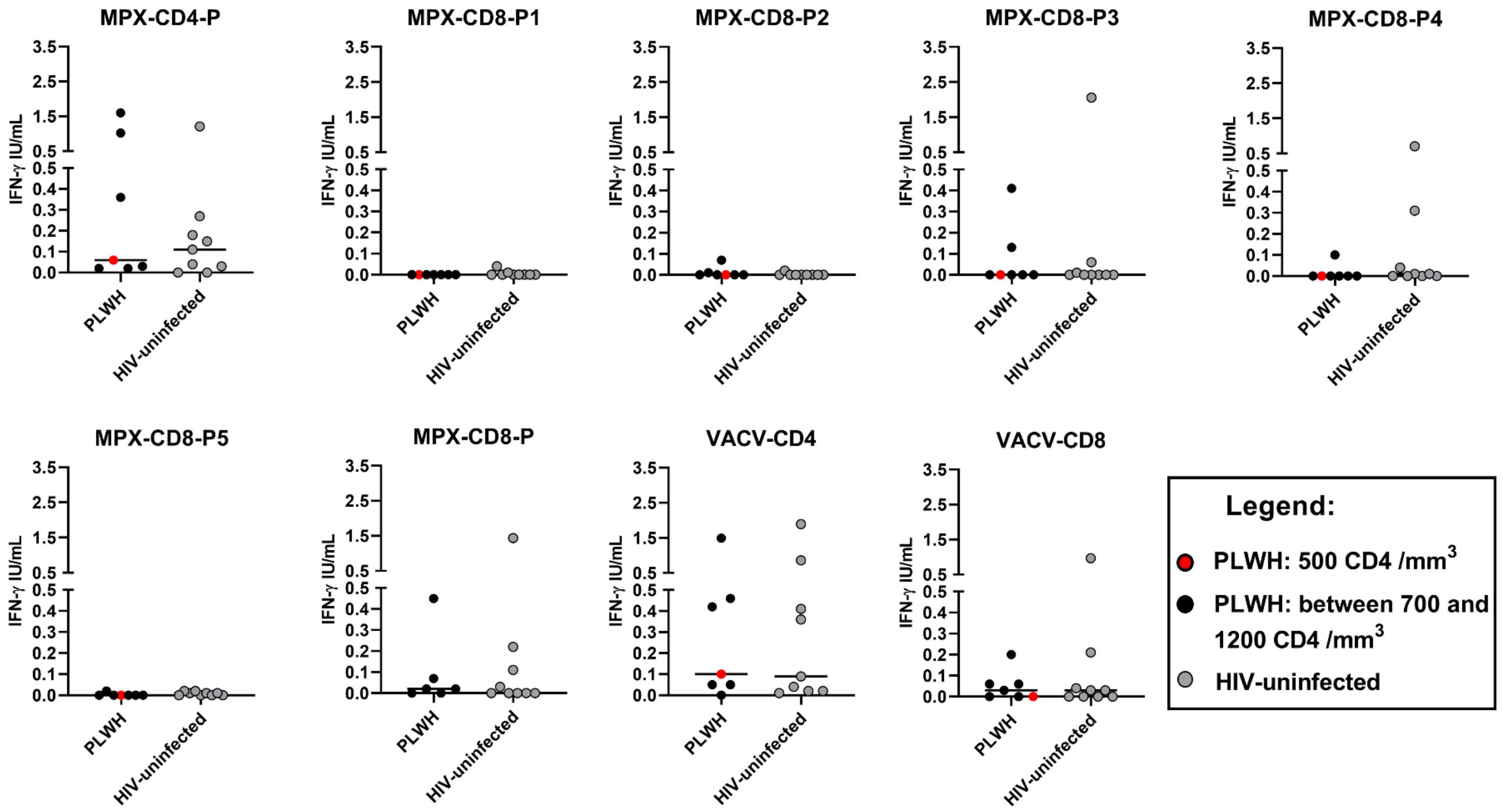

Figure S2: HIV-infection does not affect the in vitro MPXV-specific T-cell response of Mpox-cured individuals. Graphs report the IFN-γ levels in response to MPX-CD4-P; MPX-CD8-P1; MPX-CD8-P2; MPX-CD8-P3, MPX-CD8-P4, MPX-CD8-P5, MPX-CD8-P, VACV-CD4, VACV-CD8 stimulation. Results of MPX-CD8-P condition were not available for one PLWH subject. ELISA was performed in plasma samples after in vitro whole blood stimulation and IFN-γ expressed as IU/mL; the value of the stimulated condition was subtracted by the value of the unstimulated control. The horizontal lines represent the median; statistical analysis was performed using the Wilcoxon test; Footnotes: IFN-γ: interferon-γ; PLWH: people living with HIV.

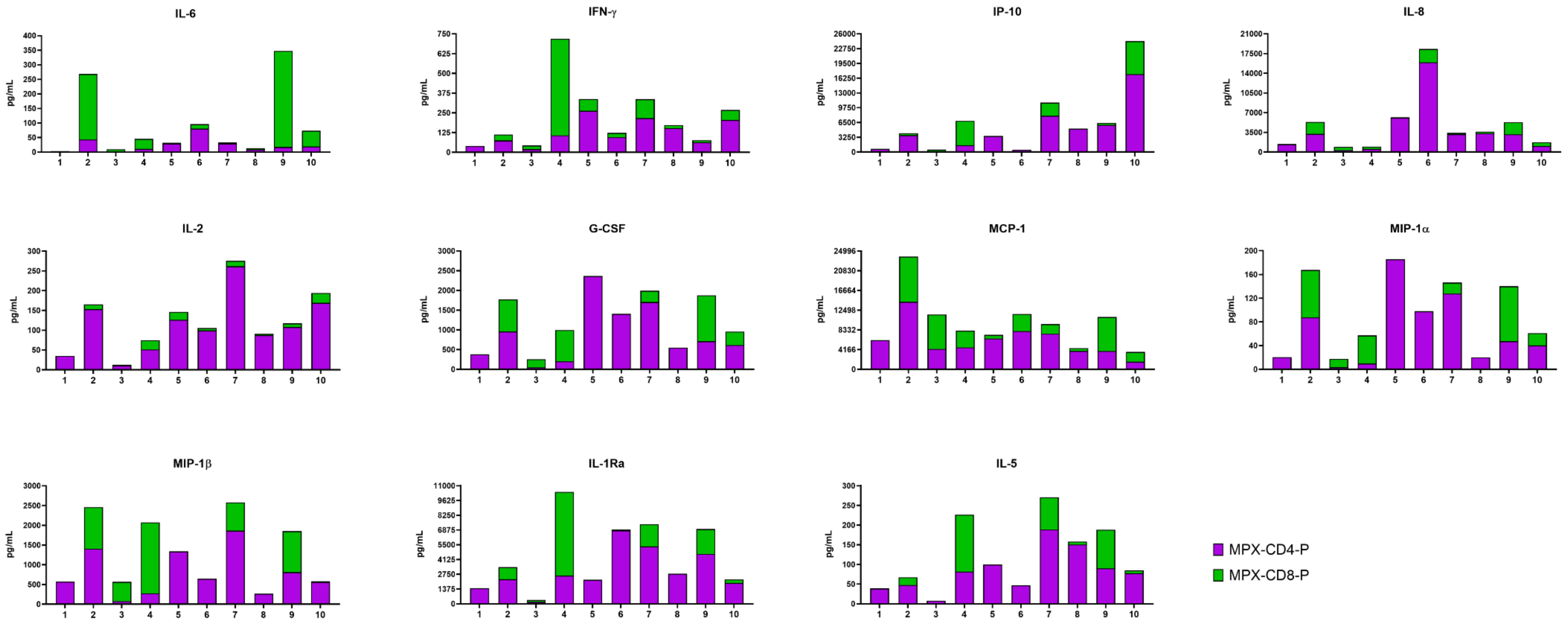

Figure S3: Mpox immune signature based on selected immune factors in Mpox-cured individuals. The graphs represent the type and quantity of the immune factors secreted in response to MPX-CD4-P, MPX-CD8-P, in each of the Mpox-cured individuals. The different immune factors were measured by luminex assay in plasma collected after in vitro stimulating whole blood with the different antigens. The value of the stimulated condition was subtracted by the value of the unstimulated control. Subject 1 was not stimulated with MPX-CD8-P. Footnotes: IFN-γ: interferon-γ; IL: interleukin; IP-10: interferon-γ inducible protein; G-CSF granulocyte- colony stimulating factor; MCP-1: monocyte chemoattractant protein-1; MIP-1: macrophage inflammatory protein-1, IL-1Ra: interleukin -1 Receptor antagonist.

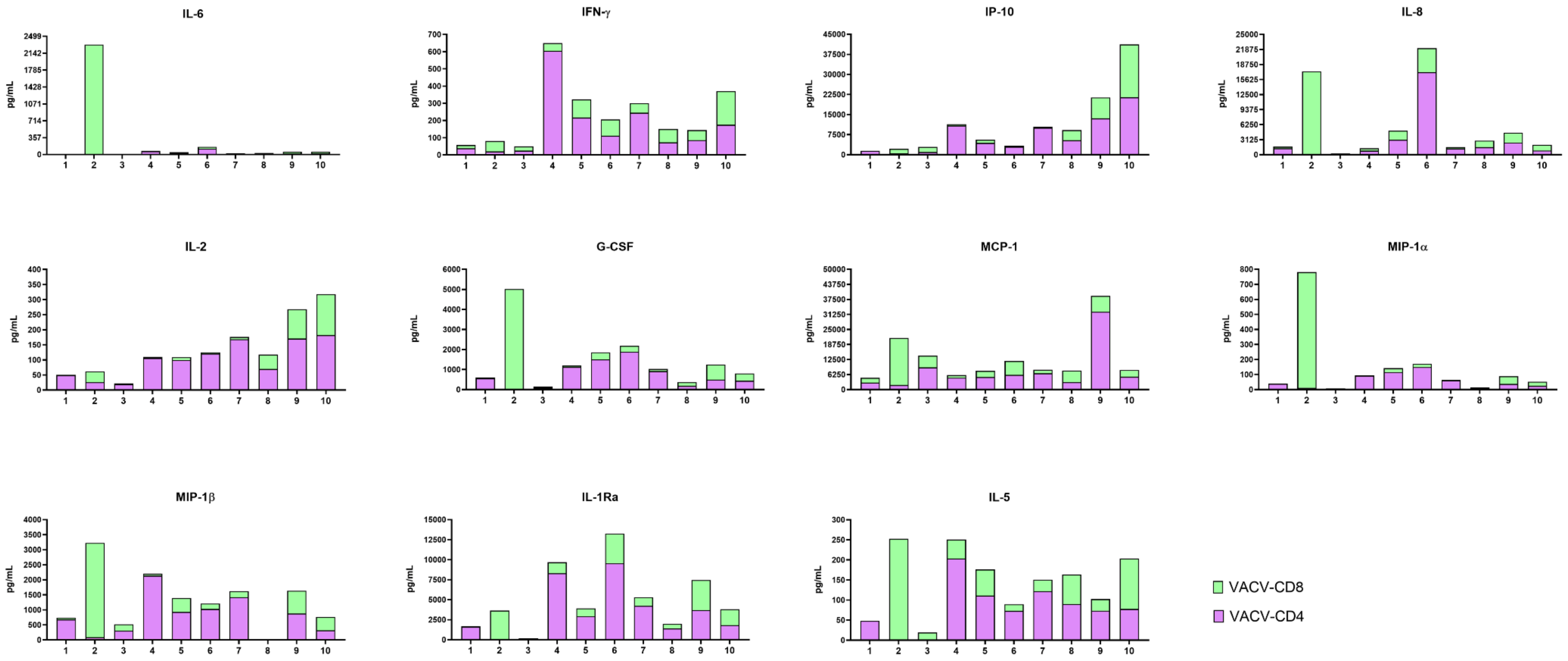

Figure S4: VACV immune signature based on selected immune factors in Mpox-cured individuals. The graphs represent the type and quantity of the immune factors secreted in response to VACV-CD4 and VACV-CD8 in each of the Mpox-cured individuals. The different immune factors were measured by luminex assay in plasma collected after in vitro stimulating whole blood with the different antigens. The value of the stimulated condition was subtracted by the value of the unstimulated control. Footnotes: IFN-γ: interferon-γ; IL: interleukin; IP-10: interferon-γ inducible protein; G-CSF granulocyte- colony stimulating factor; MCP-1: monocyte chemoattractant protein-1; MIP-1: macrophage inflammatory protein-1, IL-1Ra: interleukin -1 Receptor antagonist.

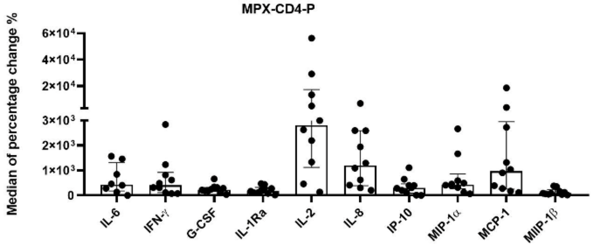

	IL-6	IFN-γ	G-CSF	IL-1Ra	IL-2	IL-8	IP-10	MIP-1α	MCP-1	MIIP-1β
25% Percentile	174.7	108.5	163.2	100.1	1120	387.4	92.02	271.2	253.9	47.54
Median	436.1	403.8	230.7	180.4	2815	1195	297.6	434.5	972.1	86.82
75% Percentile	1309	924.2	322.2	322.6	17277	2594	463.3	859.5	2956	222.6

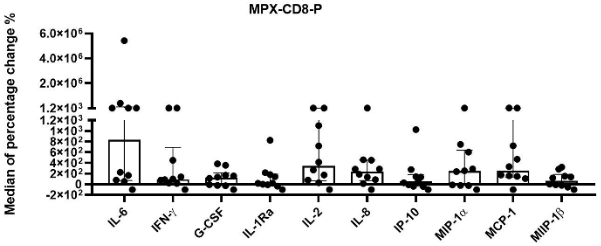

	IL-6	IFN-γ	G-CSF	IL-1Ra	IL-2	IL-8	IP-10	MIP-1α	MCP-1	MIIP-1β
25% Percentile	71.92	20.71	-24.00	-16.19	65.64	68.48	-38.54	-22.13	138.0	-26.85
Median	831.1	92.61	122.9	19.55	341.1	235.5	53.49	244.6	252.7	61.15
75% Percentile	104673	687.4	215.2	190.8	1362	453.5	177.5	638.8	1230	185.3

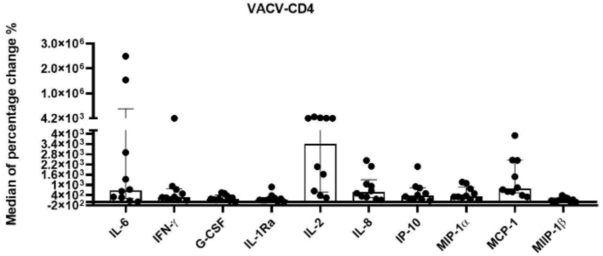

	IL-6	IFN-γ	G-CSF	IL-1Ra	IL-2	IL-8	IP-10	MIP-1α	MCP-1	MIIP-1β
25% Percentile	213.8	138.1	95.99	74.87	578.0	256.3	152.0	233.4	544.7	42.98
Median	677.6	283.0	178.8	128.7	3408	582.1	366.5	329.3	781.5	88.53
75% Percentile	388150	783.1	395.7	262.5	13202	1306	838.9	857.5	2449	228.3

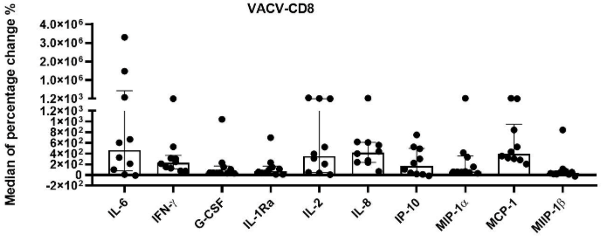

	IL-6	IFN-γ	G-CSF	IL-1Ra	IL-2	IL-8	IP-10	MIP-1α	MCP-1	MIIP-1β
25% Percentile	76.98	113.1	37.43	31.27	46.08	236.2	15.35	52.07	297.8	15.45
Median	463.6	228.0	43.05	74.89	350.6	420.0	165.3	62.59	391.7	33.89
75% Percentile	428792	365.4	167.2	165.1	5979	611.8	498.5	354.3	949.8	78.86

Figure S5: Percentage change of selected immune factors induced after in vitro Mpox stimulation in Mpx-cured individuals. The graphs represent the median percentage change calculated as: (stimulation value- unstimulation value)/unstimulation value * 100. The histograms represent the median, and the bars represent the interquartile range, the plots represent the single subject. The evaluation was not applicable if the start value was 0, for this reason, two IL-6-results e all the IL-5-results were excluded. Footnotes: IFN-γ: interferon-γ; IL: interleukin; IP-10: interferon-γ inducible protein; G-CSF granulocyte- colony stimulating factor; MCP-1: monocyte chemoattractant protein-1; MIP-1: monocyte chemoattractant protein-1, IL-1Ra: interleukin -1 Receptor antagonist.

Table S1. Antibody response to MPXV

Groups	IgM reactive N (%)	p*	IgA reactive N (%)	p*	IgG reactive N (%)	p*	Nabs reactive N (%)	p*
PLWH Cured-Mpox N=7	1 (14)	0.4375	1(14)	0.0601	7 (100)	>0.9999	7 (100)	>0.9999
HIV-uninfected Cured-Mpox N=9	0 (0)		6 (67)		9 (100)		7 (78)§	

Footnotes: IgG: immunoglobulin, Nabs: neutralizing antibodies; * Fisher's exact test; § performed on 8 subjects
